# Thoracic Spine Stenosis: Does Ultrasonic Osteotome Improve Outcome in Comparison to Conventional Technique?

**DOI:** 10.5704/MOJ.2107.010

**Published:** 2021-07

**Authors:** A Krishnan, P Samal, S Mayi, S Degulmadi, RR Rai, B Dave

**Affiliations:** Department of Spine Surgery, Stavya Spine Hospital and Research Institute, Ahmedabad, India

**Keywords:** spinal stenosis, thoracic, decompression, osteotome, ultrasonic bone scalpel

## Abstract

**Introduction:**

To investigate the efficacy of Ultrasonic Bone Scalpel (UBS) in thoracic spinal stenosis (TSS) in comparison to traditional technique.

**Material and Methods:**

A total of 55 patients who had undergone conventional surgery (Group A) are compared with 45 patients of UBS (Group B) in TSS. The primary outcome measure of Modified Japanese Orthopaedic Association score (m JOA) with neurological complications and dural injury were assessed. Secondary outcome measures of total blood loss (TBL), time duration of surgery (ORT) and length of hospital stay (LHS) were analysed.

**Results:**

The pre-operative mJOA score 5.00(4.00-6.00) in the group A and 5.00(4.00-6.00) in the group B improved to 7.00(7.00-8.00) in the group A and 9.00(9.00-10.00) in the group B, respectively (P<0.001) at final average follow-up of 117.55 months for group A and 75.69 months in group B. More significant grade of myelopathy improvement and mJOA recovery rate (RR) were noted in group B. The TBL, ORT and LHS were more favourable in group B as compared to group A (p<0.0001). The group A had 9 (16.36%) neurological deficits compared to 2 (4.44%) in group B (p<0.001). Dural tears occurred in both groups (A=11, B=9). It was more frequent and not repairable in group A but without significant statistical difference.

**Conclusion:**

UBS can reduce neurological deficits and improve outcomes in TSS. Secondarily, reduced blood loss, lesser surgical time and reduced LHS are significant added advantages of this new technology.

## Introduction

TSS with myelopathy is frequently associated with serious paralytic post-operative complications (11.5% to 33%)^1-3^. Injury due to ischemia and surgical manipulation are more likely to occur in thoracic cord^1,2^. Better clinical outcome depends on safely performing adequate neural decompression. Traditionally nibbler, osteotome and/or Kerrison rongeur were used. The foot guard entering into the already compromised neural canal repeatedly increases the chances of injury. Over the past decades, the rotating burr and the motorised high-speed drill have evolved^4^. They have been the instruments of choice and has greatly improved the neurological outcomes. It has the disadvantages of being the rotating instrument, heating, may cause dural tear and being time consuming^5,6^. Recent landmark innovation of UBS can remarkably overcome the disadvantages of conventional and burr assisted surgeries^7,8^. But there are no reported studies of UBS usage in TSS.

This study is a retrospective comparative evaluation of operated cases of TSS at our institute with conventional technique and UBS technique to test the primary hypothesis that UBS improves the immediate perioperative outcomes in terms of reduced incidence of dural injury and neurological worsening. Thus, UBS contributes to more favourable final outcome.

## Materials and Methods

This study was approved by the institutional review board of our hospital. Informed written consent was taken in all the patients. Between January 2004 and March 2014, a total 218 patients of TSS were operated at our spine centre who were reviewed retrospectively. Until 2011, we did decompression with conventional technique i.e with hand instruments only (Group A). From 2011 onward, we did decompression using ultrasonic osteotome system [Misonix Bone Scalpel, Misonix, and Farmingdale, NY, USA] (Group B).

The patients included were, TSS patients without instability, where only laminectomy and decompression procedure was done. Presence of instability and thoracic disc prolapsed or large ventral spurs lesions needing anterior decompression or fixations were excluded. Revision surgeries were excluded. Medical records and image database of these befitting patients were reviewed for details i.e. demographics including age, gender, symptoms, duration of symptoms in pre-operative phase (in weeks), severity of symptoms and neurological deficits. Late presentation was tagged to the patient if the surgery was done after 12 weeks of onset. The clinical status was further categorised according to the mJOA for thoracic myelopathy. Eleven is the highest obtainable good score in severity and 0 is the lowest score. The severity of mJOA score was graded as mild (A;8–11), moderate (B;4–7), and severe (C;0–3)^9^.

Radiological assessment consisted of evaluating the spine radiographs, magnetic resonance imaging (MRI), MR myelograph in all cases and Computed Tomography (CT) scan in few cases. The TSS diagnosis was further classified as due to ossified posterior longitudinal ligament (OPLL), ossified ligamentum flavum (OLF), small ventral spurs (VS), hypertrophied ligamentum flavum (HLF) or combination of these.

All the surgeries were performed by authors (BRD, AK). All patients were operated under general anaesthesia in prone position. Injection methyl-prednisolone 1g dose (as institute protocol) was given intra-operatively. Exposure with standard posterior midline approach was done. Blood pressure more than 100mm of Hg systolic was always maintained. No neuro-monitoring was used. In group A, laminectomy was performed with hand instruments i.e. Kerrison punches, nibbler, and/or osteotome. In group B, laminectomy was performed with the UBS. It has an assembly of an ultrasonic generator which connects to the hand-piece with a disposable titanium cutting tip as the blade/ shaver tip and irrigation tube/console. The blade works like an osteotome to make microscopic-precise cuts for enblock removal of lamina. Post-operative spinal instability was avoided by removal of only medial one-third of the facet. Layered surgical wound closure was done under a drain. If a linear dural tear was present, then it was secured with intermittent sutures and repair was performed with 4-0 Mersilk . In case of unrepairable dural avulsion fat graft /fascia graft or dural patch graft (G patch, Surgiwear, Ahmedabad, India) was put loosely and sutured. The drain was removed on the third post-operative day. Routine painkillers and antibiotics were given. From the second post-operative day, as allowed by neurological status and motivation of patient, the mobilisation was started. Patients were discharged by fifth post-operative day usually and supervised physiotherapy was continued.

The evaluation of immediate outcome was done with occurrence of neurological deficit (partial/complete) and dural injury. mJOA score and its grading severity (A, B, C) was determined before and immediate after surgery, as well as at the final follow-up visit. Post-operative neurological recovery was estimated on the basis of the recovery rate (RR) = (post-operative - pre-operative mJOA score) / (11-pre-operative mJOA score) *100%. A score of 75–100% was designated as excellent, 50–74% good, 25–49% fair, and 0–24% poor^9^. Additionally, total number of segment levels operated, ORT (minutes), time taken per segment of laminectomy, TBL including intra operative blood loss (IOBL), post-operative blood loss in drain (POBL), visualised epidural bleeding and blood loss per segment of laminectomy and LHS were assessed. Intra-operative complications like dural thermal burns as visible blackening of dura (UBS only), location/pattern of dural tear (linear or avulsion), its management, inadvertent over-cutting leading to spinal instability and any visible neural injury in both groups were noted. The data, patient follow-up and images were collected and tabulated by surgeons who were not part of operating surgeon's team to avoid bias. If the old images were deficient then the previous radiological case reports were endorsed by consensus. When old images were not found for reconfirmation, then the interpretations entered in operative note and case record were considered optimum.

Analysis of the patient’s demographics and characteristic categorical variables were done. Mean (SD: Standard Deviation) for applicable variables were calculated. Median (Inter-quartile range: IQR) were used for outcome parameters which were non-parametric. When data was not normally distributed each category was compared by using suitable statistical tools such as Wilcoxon Test(W), Mann Whitney test(U) and Chi-square test(X^2^) for non-parametric data & unpaired ‘t’ test was used for parametric data. If the data was not normally distributed then non-parametric test values were used for comparison. Normality was checked using Shapiro Wilks test. The significance of the relation was considered in patients only if p<0.05. The software used is SPSS version 20.0.

## Results

The study comprised of a total of 100 patients of TSS, who were operated and met the inclusion criteria. Out of the 218 total patients, 118 who had stenosis due to infection, trauma, disc prolapse or ventral stenosis with or without instrumented fusion were excluded. There was no significant difference in most demographic variables, sign/symptoms, types of lesion and levels of pathology between both groups (Table I, p value >0.05). So, both the groups were comparable and homogenous. The study variables were analysed and summarised.

**Table I:** Comparison of demographic, clinical feature and radiological variables in the two groups

Variables	Group A	Group B	p-Value
**Demography**			
No. of patients	55	45	-
Male/Female	24/31	26/19	-
BMI	28.56^a^ (7.97) ^b^	29.37^a^ (9.38) ^b^	0.967
Age (Years)	53.51^a^ (12.24) ^b^	56.33^a^ (11.63) ^b^	0.243
Presenting symptom duration (Weeks)	16.01^a^ (15.59) ^b^	13.52^a^ (10.54) ^b^	0.001
**Symptoms / Signs: Number of patients (%)**			
Back pain	40(72.72%)	37(82.22%)	1.25
Leg pain (lower limb symptoms)	43(78.18%)	30(66.66%)	1.34
Vesicular dysfunction	20(36.36%)	15(33.33%)	0.319
Power <3 in at least 1 group of muscles ^c^	14(25.4%)	17(37.78%)	1.33
mJOA	5^d^ (4.00 - 6.00)^e^	5^d^ (4.00 - 6.00)^e^	0.335
**Radiological Variables**			
**Type of lesions: Number of patients**			
OLF or thickened ligamentous flavum	54	42	0.473
Facetal arthritis: spondylosis (hypertrophy)	11	9	0.99
OPLL	3	1	0.758
Ventral spur	4	5	0.752
OPLL+OLF	3	1	0.758
**Levels: Number of patients (%)**			
Upper (T1-4)	2(3.64%)	3(6.67%)	0.818
Middle (T5-8)	5(9%)	4(8.89%)	0. 972
Lower (T9-T12)	48(87.27%)	38(84.44%)	0.894

Abbreviations; OLF: Ossified Ligament Flavum, OPLL: Ossified Posterior longitudinal Ligament Flavum, T: Thoracic, ^a^Mean : ^b^ SD (Standard Deviation), ^d^Median : ^e^ IQR (Inter Quartile Range) ^c^MRC (Medical Research council) grading

The demography, clinical features (symptoms / signs) and radiological features are tabulated (Table I). Late presentation(n=29) was noted in 16 and 13 patients in group A and group B respectively. In both the groups T10-11 vertebral level was most commonly affected (group A: n=18, and group B: n= 14). The myelopathy classification as per mJOA score was graded as mild (A; n=2) moderate (B; n=44), and severe (C; n=9) in group A. In group B it was graded as mild (A; n=4) moderate (B; n=38), and severe (C; n=3). The grade of myelopathy in both group patients combined together improved from grade C(n=12) to A(n=5,41.66%), grade B(n=81) to A(n=57,70.37%) and others remained same grade A(n=7,100%). Though, there was no statistical significance between the two groups. The mean ORT (skin to skin), time taken per segment, IOBL, POBL, TBL and TBL per segment and LHS were more significantly favourable in group B as compared to group A (Table II). The mean follow-up of group A was 117.55(24.60) months and for group B was 75.69(6.07) months. The mJOA score improved in both the groups A and B combined significantly (W=8.614, p<0.001) at final follow-up. In group A pre-operative mJOA improved from 5(4.00-6.00) to 7(7.00-8.00) (W=5.88, P<0.001). Whereas in group B pre-operative mJOA improved from 5(4.00-6.00) to 9(9.00-10.00) (W=6.38, P<0.001). But, in group B there was more significant grade of improvement as compared to group A. The minimum clinically important difference was much significant in group B. The mJOA recovery rate also in the group B was more favourable (Table III) (p<0.001). There were 3 patients of severe grade C mJOA who improved to grade A in group B (100%). But, in group A, out of 9 patients of severe grade C mJOA, only 2 patients improved to grade A (22.22%) and other 7(77.77%) improved to grade B. Though, this was statistically insignificant within the groups (group A, p=0.706; group B, p=0.08). But it is notable that in RR there are no excellent results in group A, though group B has excellent results in 17(37.80%) patients. Also, the group B has no poor result, though group A has poor results in 6(10.90%) patients. There was no significant mJOA outcome difference in patients presenting late or before 3 months.

**Table II:** Comparison of secondary surgical outcome variables between the two groups

Surgical events	Group A (Conventional) Median (IQR)	Group B (Bone Scalpel) Median (IQR)	P value
Level decompression (segments)	2.0 (2.0-3.0)	3.0(2.0-4.0)	0.228
Total Operative time (minutes)	140.0(140.0 - 160.0)	100.0(90.0 - 115.0)	<0.0001
Decompression/segment time (minutes)	55.0(45.0-70.0)	38.0(30.0-43.0)	<0.0001
Blood loss (IOBL) (ml)	350.0(320.0-380.0)	250.0(200.0-295.0)	<0.0001
POBL (ml)	325.0(300.0-410.0)	310.0(277.50-320.0)	<0.0001
TBL segment(ml)	135.0(103.0-180.0)	90.0(67.0-120.0)	<0.0001
TBL (ml)	690.0 (610.01-770.0)	540.0(485.0-620.0)	<0.0001
LHS (days)	5.0 (4.0-6.0)	4.0(4.0-4.50)	<0.0001

Abbreviations; IOBL: Intra Operative Blood Loss, POBL: Post Operative Blood Loss, TBL: Total Blood Loss, LHS: Length of Hospital Stay

**Table III:** Comparison of mJOA recovery rate between the two groups

Recovery rate	Group A	Group B
Excellent (75-100)	0(0%)	17(37.80%)
Good (50 - 74)	21(38.20%)	24(53.30%)
Fair (25 - 49)	28(50.90%)	4(8.90%)
Poor (0-24)	6(10.90%)	0(0%)
	X^2^=40.60	p<0.001

There was remarkable epidural bleed in all patients of group A and only in 7 patients of group B. There was notable absence of bleed from exposed ends of the bone in group B. Patients had variable frequency of dural injury (tear or avulsion) occurrence and their different patterns in both the groups (Group A=11, Group B=9; p=0.8594). In the group A, the dural injuries were dural tears due to direct biting with the Kerrison rongeurs (n=5) and avulsion injuries (n=6), while removing the adherent ossified part. All of these were non-repairable. In group B, the dural injuries were due to the UBS blade itself (n=3) which occurred during the lamina segment osteotomy. These tears tended to be linear in quality and lateral in location and cranio-caudal in orientation. These occurred at corners, where it takes longer time to complete the osteotomy. As they were more linear in nature, they were easily repairable. In rest of the patients (n=6) in group B, the dural injury was due to avulsion of adherent ossified dura. After completed osteotomy, as the cut bone of lamina was en-block lifted off, the dura had got avulsed. Repair was not possible in these cases.

Neurological deficit invariably occurred in both groups with more preponderance in group A (n=9) as compared to group B (n=2). Of the 9 worsened patients in group A, 1 patient had transient worsening and he recovered completely within 48 hours. Three patients had complete paraplegia with bladder/bowel involvement. Five patients had incomplete worsening. All of whom improved at 12 weeks and bettered than pre-operative mJOA at the final follow-up. The neurological worsening in group A or B was not correlating with the severe (grade C) of pre-operative mJOA but was found to be more in grade B of myelopathy. No reason there off could be related to it. Of the 2 patients who had neurological deficit in group B, one patient had pre-operative spastic paraplegia (non-walker for 1.5 years, mJOA=4). But, after surgery bladder and bowel also got affected. Second patient had neurological deficit post-operatively and his preoperative mJOA was 4. They both recovered to mJOA 4 and 7, respectively at final follow-up. The deterioration of neurology in Group A was from median 6(4.50-7.00) to 2(2.00-3.00) (W=2.682, P=0.007). This was significant. The deterioration of neurology in Group B was from median 4 to 1(IQR not applicable, as 2 observations only) (W=1.342, P=0.18). It was not significant. There was more severe grade of deterioration noted in Group A. In group A, there were 4 (54.50%) patients with both neurological deficit and dural tears. Whereas 5 (45.50%) patients had only neurological deficit. Whereas both the patients of group B had dural injury. These patients in both groups didn’t show any significant pattern of neurological recovery when compared to those who didn’t have a dural injury.

For both groups, average duration of LHS in those having dural injury and neurological deficit(n=24) was 6.21(1.35) days. But when compared to those who didn’t have dural injury and neurological deficit(n=76), they had an average 4.46(0.87) days of LHS. Overall LHS was related to neurological deficit and/or dural tear and its repercussions (p<0.001). In Group A the average LHS was 6.50(1.36) day. Whereas in group B, the average LHS was 5.63(1.18) days. Though LHS was more in group A, but it was non-significant statistically(p=0.13) when compared to group B. No other complications of iatrogenic instability were noted. There were no reoperations till final follow-up. Three patients in group A and 4 in group B had urinary tract infection, which were conservatively managed. Some patients developed positional headaches, nausea and vomiting post-operatively (Group A: n=3, Group B: n=4). Hence, they needed delayed ambulation and symptomatic care.

## Discussion

Degenerative disorders like intervertebral disc herniation, OLF, OPLL and VS causing TSS is not uncommon^10^. Primary reason for severity of TSS is late and misdiagnosis^11^. The most important predictor of a good post-operative outcome is a less affection of pre-operative mJOA score^12-14^. In our study it was also noted that mild and moderate grade myelopathy patients have better follow-up mJOA. In our series also late presentation was noted in 29%. The mJOA improvement in both groups combined was significant grade of improvement after surgery. The mJOA improvement in group B was more as compared to group A(p<0.001). The RR was also noted to be more significant in group B(p<0.001).

Low decompressive range, direct surgical insult and re-perfusion injury are common causes of iatrogenic spinal cord injury^15-18^. The thoracic spinal cord has a more precarious blood supply and the region of watershed extends from T2 to T719. We can only assume the possible reasons for acute neurological complications which occur after surgery is done. Possibly the surgical intervention plays role in neurological deterioration due to manipulation and irritation of the cord^20^. Overall (combined group A and B), neurological deterioration in our series (n=11,11%) is low and on the comparable better side of literature (11.7 to 33%)^1,3,10,11,15^. The group A ended with more number (n=9,16.36%) of neurological deficit than group B (n=2,4.44%) (p<0.001).

**Fig. 1:**
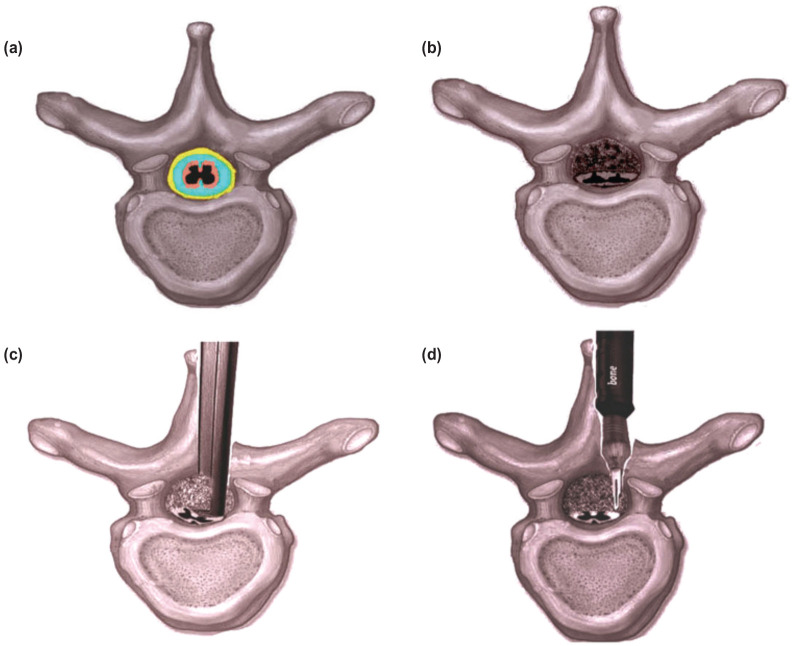
Illustration showing (a) Normal thoracic cord cushioned by cerebrospinal fluid and fat. (b) Established stenosis with loss of the natural margin of safety of the fluid and fat. (c) Kerrison rongeur foot plate enters and compresses the cord tissue directly. (d) UBS does least violation while cutting the lamina and stenosing tissue.

**Fig. 2:**
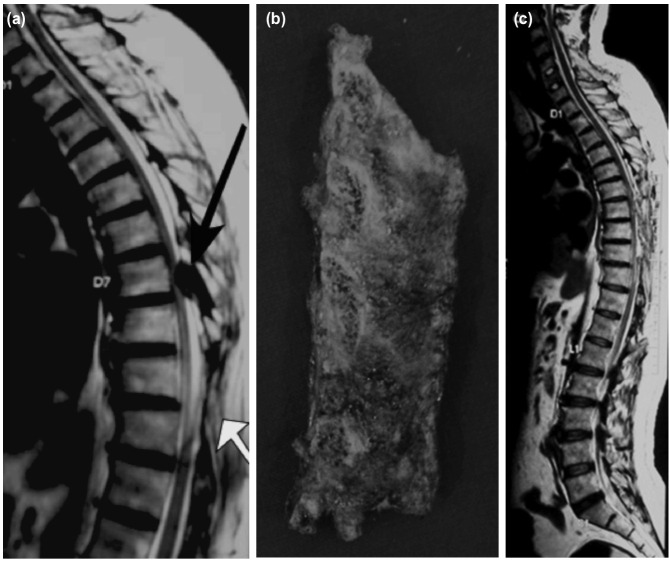
At 2-year follow-up of a patient, operated for T9-10 stenosis with conventional method developed a new stenosis at T7-8. The 1st ORT was 90 minutes and a post-operative deficit occurred. It was complete paraplegia and then recovered slowly beyond a pre-operative mJOA of 5 to final mJOA of 9 at 12 months. At the 2nd presentation of the same patient, the mJOA score again deteriorated to 6. Second surgery was with UBS and with a quick ORT of 55 minutes. There was no post-operative deficit in second surgery, mJOA again improved to 9 and maintained at 5-year follow-up. (a) T2 sagittal MRI (white arrow showing operated laminectomy and black arrow showing new stenosis). (b) En-block laminectomy bone removed with bone scalpel (c) Post-operative T2 sagittal MRI showing adequate decompression.

The high-speed drill burr can possibly directly injure the cord or cause heat injury^4^. The burr may also catch hold of cotton pledgets (grabbing), which necessitates interruption. Also, there is need for frequent irrigation and suction. To thin out lamina and facets the burr is used. But the actual tearing mechanism is due to Kerrison rongeur and from accessory instruments used during final decompression. Introduction of a Kerrison rongeur footplate underneath stenotic bony elements may results in spinal cord maceration and irritation of cord and its blood supply (Fig. 1a, b, c). Irrigation is must for the burr; or else it leads to scorching of bone^5^. Though, due care is taken during microsurgical techniques, the risk of injuring adjacent sensitive structures exists with unexpected neurological deficits. But, the incidence of deterioration has reduced as compared to use of hand instruments^8^. UBS is novice in spine surgery. It offers greater precision in bone cutting and decreases chances of collateral damage^7,8,21^. Piezo-surgery, is based on ultrasonic micro-vibrations, that allows not only selective cutting of the bone but also reserving the critical soft tissues^21^. Theoretically, the UBS does not have risk of dural and neural injury (Fig. 1d). The UBS uses a self-irrigating system that provides cooling, limiting mechanical and thermal injury risks^8,22,23^. We also observed a major advantage which was though difficult to objectively quantify. It was noted that there was a nearly absent cut surface bone bleeding and reduced epidural bleeding when compared with group A. Thus, the blood loss was reduced remarkably. This has been confirmed in ovines as well^8^. This was proved in our study with the significant reduction of TBL, TBL per segment, IOBL and POBL (p<0.001) in group B. The UBS can also significantly shorten the ORT required for the decompression compared to use of conventional method and this was also statistically significant (p<0.001) (Fig. 2).

In all cases of dural injury in group A, the actual reason for injury was use of the Kerrison rongeur. In dural tears of the UBS, the tear was due to the blade itself, and not from accessory instruments. In group B tears tended to be more linear and cranio-caudal orientated in comparison to group A dural tears. They were laterally located and repairable. In this study the dural tear due to UBS direct burn injury incidences were 6.66% (n=3) and were noticed in early learning experience. Sometime the dural calcification or adherence makes dural avulsion inevitable. So, avulsion did occur in group B (n=6) as well due to adherent pathology.

Late diagnosis till severe stenosis is also one of the reasons for more post-operative deficits^2,10,16^. Group A ended with more (n=9,16.4%) neurological deficits than group B (n=2,4.5%). The neurological deficits though occurred in both groups, it was noted more in group A. Moreover, the severity of deterioration was also more pronounced in group A. This signifies that the UBS may cause dural injury but still is relatively safe for neural tissue. The recovery of the patients with dural tear or neurological deficit was unrelated to those who had no deficit or tear in both the groups. The LHS was also less in group B which is clinically related to neural deficits, dural injury and its ramifications.

There are many limitations of our study due to its retrospective nature. The group was not completely homogenous on the basis of presenting symptoms duration and sex ratio. Moreover, the UBS was compared with hand mechanical instruments and not with the current literature standard of burr. We in our learning curve progressed from hand instruments to UBS and there was no major burr experience in thoracic spine surgery. The neurological outcome, bleeding and operation time reduction though are more favourable in the group B and learning experience and surgical skill improvement of surgeons may have also immensely contributed to the better outcomes and not analysed in current study. The cost of technology also not assessed. But there is at present no comparative literature study showing the better utility of UBS and this study with its limitations shows its productive application. The bleeding observed from cut ends of bone and epidural space also cannot be quantified in the present study and is just a visual observation. Though, it is mentioned in previous literature as well. As we realised the huge benefits of UBS, we never attempted to use the burr in this group of surgeries from outset. Creating now a group to compare the burr and UBS was unethical. Therefore, further scope of additional prospective studies involving more than single institutions are needed to clarify the efficacy and utility of UBS.

## Conclusion

Surgical decompression of TSS is a challenging procedure with further deterioration of the neurology being quite frequent in conventional methods. To improve outcome and avoid neurological worsening, UBS is a safer alternative. Reduced LHS, blood loss and surgical time are added advantages of the technology.
